# 2,3-Diamino­pyridinium 4-carb­oxy­butano­ate

**DOI:** 10.1107/S1600536811044473

**Published:** 2011-10-29

**Authors:** Madhukar Hemamalini, Jia Hao Goh, Hoong-Kun Fun

**Affiliations:** a X-ray Crystallography Unit, School of Physics, Universiti Sains Malaysia, 11800 USM, Penang, Malaysia

## Abstract

In the title mol­ecular salt, C_5_H_8_N_3_^+^·C_5_H_7_O_4_^−^, the 2,3-diamino­pyridine mol­ecule is protonated at the pyridine N atom. The cation is essentially planar, with a maximum deviation of 0.015 (2) Å, and the anion adopts an extended conformation. In the crystal, the hydrogen glutarate (4-carb­oxy­butano­ate) anions are self-assembled through O—H⋯O hydrogen bonds, forming chains. The cations are connected to the anion chains *via* N—H⋯O hydrogen bonds, forming a three-dimensional network. The crystal structure also features aromatic π–π inter­actions between the pyridinium cations, with a centroid–centroid distance of 3.4464 (10) Å.

## Related literature

For applications of 2-amino­pyridine derivatives, see: Bis *et al.* (2006 ▶); Gellert & Hsu (1988 ▶). For glutaric acid conformations, see: Saraswathi *et al.* (2001 ▶). For hydrogen-bond motifs, see: Bernstein *et al.* (1995 ▶). For bond-length data, see: Allen *et al.* (1987 ▶). For the stability of the temperature controller used in the data collection, see: Cosier & Glazer (1986 ▶).
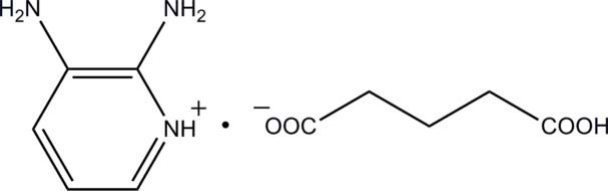


## Experimental

### 

#### Crystal data

C_5_H_8_N_3_^+^·C_5_H_7_O_4_^−^
*M* *_r_* = 241.25
Monoclinic, 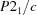

*a* = 7.7052 (1) Å
*b* = 21.4626 (4) Å
*c* = 7.8450 (1) Å
β = 119.473 (1)°
*V* = 1129.46 (3) Å^3^
*Z* = 4
Mo *K*α radiation
μ = 0.11 mm^−1^
*T* = 100 K
0.35 × 0.18 × 0.05 mm

#### Data collection

Bruker APEXII DUO CCD diffractometer
Absorption correction: multi-scan (*SADABS*; Bruker, 2009 ▶) *T*_min_ = 0.962, *T*_max_ = 0.994
9826 measured reflections
3281 independent reflections
2475 reflections with *I* > 2σ(*I*)
*R*_int_ = 0.030

#### Refinement

*R*[*F* ^2^ > 2σ(*F* ^2^)] = 0.050
*wR*(*F* ^2^) = 0.123
*S* = 1.04
3281 reflections
191 parameters
H atoms treated by a mixture of independent and constrained refinement
Δρ_max_ = 0.43 e Å^−3^
Δρ_min_ = −0.35 e Å^−3^

### 

Data collection: *APEX2* (Bruker, 2009 ▶); cell refinement: *SAINT* (Bruker, 2009 ▶); data reduction: *SAINT*; program(s) used to solve structure: *SHELXTL* (Sheldrick, 2008 ▶); program(s) used to refine structure: *SHELXTL*; molecular graphics: *SHELXTL*; software used to prepare material for publication: *SHELXTL* and *PLATON* (Spek, 2009 ▶).

## Supplementary Material

Crystal structure: contains datablock(s) global, I. DOI: 10.1107/S1600536811044473/hb6458sup1.cif

Structure factors: contains datablock(s) I. DOI: 10.1107/S1600536811044473/hb6458Isup2.hkl

Additional supplementary materials:  crystallographic information; 3D view; checkCIF report

## Figures and Tables

**Table 1:** Hydrogen-bond geometry (Å, °)

*D*—H⋯*A*	*D*—H	H⋯*A*	*D*⋯*A*	*D*—H⋯*A*
N1—H1*N*1⋯O2^i^	0.86	1.94	2.7571 (18)	159
N2—H2*N*1⋯O1^i^	0.86	2.13	2.9077 (19)	151
N2—H2*N*2⋯O1^ii^	0.86	2.04	2.8766 (18)	164
N3—H3*N*1⋯O4^iii^	0.86	2.16	3.0054 (18)	168
N3—H3*N*2⋯O1^ii^	0.86	2.17	3.0194 (18)	167
O3—H1*O*1⋯O2^iv^	0.82	1.74	2.5546 (18)	171

Symmetry codes: (i) 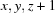; (ii) 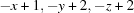; (iii) 
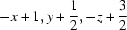
; (iv) 
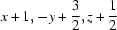
.

**Fig. 1.**
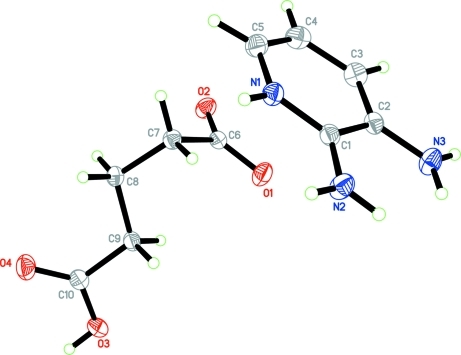
The asymmetric unit of the title compound, showing 50% probability displacement ellipsoids.

**Fig. 2.**
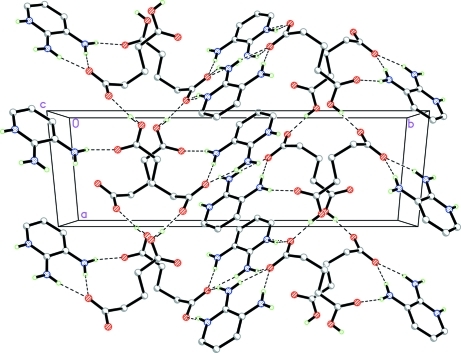
The crystal packing of title compound (I).

## supplementary crystallographic information

### Comment

2-Aminopyridine and its derivatives are some of the most frequently-used synthons in supramolecular chemistry based on hydrogen bonds (Bis *et al.*, 2006; Gellert & Hsu, 1988).  Glutaric acid is found in the blood and urine. It is used in the synthesis of pharmaceuticals, surfactants and metal finishing compounds.  Herein, we report the crystal structure determination of the title compound, (I).

The asymmetric unit (Fig. 1) contains a 2,3-diaminopyridinium cation and hydrogenglutarate anion. The cation is essentially planar, with a maximum deviation of 0.015 (2) Å for atom C1. In the 2,3-diaminopyridinium cation, a wide angle [123.94 (14)°] is subtended at the protonated N1 atom. The conformation of the hydrogenglutarate anion can be described by the two torsion angles C6-C7-C8-C9 of  58.61 (16)° and C7-C8-C9-C10 of 175.91 (13)°. As evident from the torsion angles, the hydrogenglutarate anion is in a fully extended conformation (Saraswathi *et al.*, 2001). Of the two carboxyl groups, one is deprotonated while the other is not. The bond lengths (Allen *et al.*, 1987) and angles are within normal ranges.

In the crystal (Fig. 2), the protonated N1 atom and the 2-amino group (N2) are hydrogen-bonded to the carboxylate oxygen atoms (O1 and O2) *via* a pair of intermolecular N—H···O hydrogen bonds, forming a ring motif *R*^2^_2_(8) (Bernstein *et al.*, 1995). The hydrogen glutarate anions self-assemble through O—H···O hydrogen bonds, forming chains. Furthermore, the cations are connected via N—H···O hydrogen bonds (Table 1) to these anoinic chains to form a three-dimensional network. The crystal structure is further stabilized by weak π–π interactions between the pyridinium (Cg1 = N1/C1–C5) cations [Cg1···Cg1 = 3.4464 (10) Å; -x, 2-y, 2-z].

### Experimental

Hot methanol solution (20 ml) of 2,3-diaminopyridine (52 mg, Aldrich) and glutaric acid (66 mg, Merck) were mixed and warmed over a heating magnetic stirrer hotplate for a few minutes. The resulting solution was allowed to cool slowly at room temperature and brown plates of the title compound appeared after a few days.

### Refinement

The C-bonded hydrogen atoms were located from a difference Fourier maps and refined freely [C–H = 0.96 (2)–1.00 (2) Å] and C–H = 0.93 (2)–1.01 (2) Å]. The O- and N- bonded hydrogen atoms can also be located but in the final refinement, these hydrogen were positioned geometrically [N–H = 0.86 Å and O–H = 0.82°] and were refined using a riding model, with *U*_iso_(H) = 1.2 or 1.5 *U*_eq_(C).

### Crystal data

**Table:**

C_5_H_8_N_3_^+^·C_5_H_7_O_4_^−^	*F*(000) = 512
*M**_r_* = 241.25	*D*_x_ = 1.419 Mg m^−^^3^
Monoclinic, *P*2_1_/*c*	Mo *K*α radiation, λ = 0.71073 Å
Hall symbol: -P 2ybc	Cell parameters from 3028 reflections
*a* = 7.7052 (1) Å	θ = 3.1–30.0°
*b* = 21.4626 (4) Å	µ = 0.11 mm^−^^1^
*c* = 7.8450 (1) Å	*T* = 100 K
β = 119.473 (1)°	Plate, brown
*V* = 1129.46 (3) Å^3^	0.35 × 0.18 × 0.05 mm
*Z* = 4	

### Data collection

**Table:**

Bruker APEXII DUO CCD diffractometer	3281 independent reflections
Radiation source: fine-focus sealed tube	2475 reflections with *I* > 2σ(*I*)
graphite	*R*_int_ = 0.030
φ and ω scans	θ_max_ = 30.1°, θ_min_ = 3.0°
Absorption correction: multi-scan (*SADABS*; Bruker, 2009)	*h* = −10→9
*T*_min_ = 0.962, *T*_max_ = 0.994	*k* = −30→16
9826 measured reflections	*l* = −11→10

### Refinement

**Table:**

Refinement on *F*^2^	Primary atom site location: structure-invariant direct methods
Least-squares matrix: full	Secondary atom site location: difference Fourier map
*R*[*F*^2^ > 2σ(*F*^2^)] = 0.050	Hydrogen site location: inferred from neighbouring sites
*wR*(*F*^2^) = 0.123	H atoms treated by a mixture of independent and constrained refinement
*S* = 1.04	*w* = 1/[σ^2^(*F*_o_^2^) + (0.0439*P*)^2^ + 0.6889*P*] where *P* = (*F*_o_^2^ + 2*F*_c_^2^)/3
3281 reflections	(Δ/σ)_max_ = 0.001
191 parameters	Δρ_max_ = 0.43 e Å^−^^3^
0 restraints	Δρ_min_ = −0.35 e Å^−^^3^

### Special details

**Table:**

Experimental. The crystal was placed in the cold stream of an Oxford Cryosystems Cobra open-flow nitrogen cryostat (Cosier & Glazer, 1986) operating at 100.0 (1) K.
Geometry. All s.u.'s (except the s.u. in the dihedral angle between two l.s. planes) are estimated using the full covariance matrix. The cell s.u.'s are taken into account individually in the estimation of s.u.'s in distances, angles and torsion angles; correlations between s.u.'s in cell parameters are only used when they are defined by crystal symmetry. An approximate (isotropic) treatment of cell s.u.'s is used for estimating s.u.'s involving l.s. planes.
Refinement. Refinement of F^2^ against ALL reflections. The weighted R-factor wR and goodness of fit S are based on F^2^, conventional R-factors R are based on F, with F set to zero for negative F^2^. The threshold expression of F^2^ > 2σ(F^2^) is used only for calculating R-factors(gt) etc. and is not relevant to the choice of reflections for refinement. R-factors based on F^2^ are statistically about twice as large as those based on F, and R- factors based on ALL data will be even larger.

### Fractional atomic coordinates and isotropic or equivalent isotropic displacement parameters (Å^2^)

**Table:**

	*x*	*y*	*z*	*U*_iso_*/*U*_eq_	
N1	0.1547 (2)	0.91493 (6)	1.0497 (2)	0.0199 (3)	
H1N1	0.1763	0.8895	1.1428	0.024*	
N2	0.4178 (2)	0.97294 (6)	1.27871 (19)	0.0227 (3)	
H2N1	0.4358	0.9454	1.3657	0.027*	
H2N2	0.4941	1.0051	1.3105	0.027*	
N3	0.3437 (2)	1.06274 (6)	0.9866 (2)	0.0236 (3)	
H3N1	0.3206	1.0897	0.8967	0.028*	
H3N2	0.4382	1.0690	1.1044	0.028*	
C1	0.2710 (2)	0.96571 (7)	1.0940 (2)	0.0174 (3)	
C2	0.2301 (2)	1.00984 (7)	0.9422 (2)	0.0179 (3)	
C3	0.0765 (3)	0.99662 (8)	0.7572 (2)	0.0218 (3)	
C4	−0.0356 (3)	0.94140 (8)	0.7180 (2)	0.0243 (3)	
C5	0.0045 (2)	0.90133 (8)	0.8661 (2)	0.0230 (3)	
O1	0.37775 (18)	0.91001 (5)	0.58579 (17)	0.0259 (3)	
O2	0.14921 (17)	0.85057 (5)	0.35117 (16)	0.0231 (3)	
O3	0.89545 (17)	0.73680 (5)	0.77123 (18)	0.0250 (3)	
H1O1	0.9696	0.7065	0.8003	0.037*	
O4	0.68383 (19)	0.66708 (5)	0.78111 (19)	0.0286 (3)	
C6	0.2633 (2)	0.86410 (7)	0.5313 (2)	0.0184 (3)	
C7	0.2679 (2)	0.81908 (7)	0.6837 (2)	0.0180 (3)	
C8	0.3856 (2)	0.76040 (7)	0.6920 (2)	0.0177 (3)	
C9	0.5983 (2)	0.77618 (7)	0.7421 (2)	0.0181 (3)	
C10	0.7278 (2)	0.72033 (7)	0.7669 (2)	0.0190 (3)	
H3A	0.044 (3)	1.0263 (9)	0.654 (3)	0.031 (5)*	
H4A	−0.143 (3)	0.9321 (9)	0.589 (3)	0.031 (5)*	
H5A	−0.066 (3)	0.8624 (9)	0.854 (3)	0.026 (5)*	
H7A	0.331 (3)	0.8398 (8)	0.812 (3)	0.019 (4)*	
H7B	0.131 (3)	0.8081 (9)	0.649 (3)	0.025 (5)*	
H8A	0.317 (3)	0.7388 (8)	0.562 (3)	0.019 (4)*	
H8B	0.389 (3)	0.7319 (8)	0.792 (3)	0.018 (4)*	
H9A	0.601 (3)	0.8033 (9)	0.642 (3)	0.025 (5)*	
H9B	0.664 (3)	0.8000 (9)	0.867 (3)	0.028 (5)*	

### Atomic displacement parameters (Å^2^)

**Table:**

	*U*^11^	*U*^22^	*U*^33^	*U*^12^	*U*^13^	*U*^23^
N1	0.0211 (6)	0.0171 (6)	0.0228 (7)	−0.0007 (5)	0.0118 (5)	0.0011 (5)
N2	0.0241 (7)	0.0215 (6)	0.0182 (7)	−0.0050 (5)	0.0071 (6)	0.0039 (5)
N3	0.0300 (7)	0.0199 (6)	0.0181 (6)	−0.0049 (5)	0.0097 (6)	0.0028 (5)
C1	0.0182 (7)	0.0157 (7)	0.0196 (7)	0.0011 (5)	0.0102 (6)	0.0001 (5)
C2	0.0208 (7)	0.0160 (6)	0.0192 (7)	0.0014 (5)	0.0117 (6)	0.0006 (5)
C3	0.0251 (8)	0.0238 (8)	0.0168 (7)	0.0010 (6)	0.0105 (6)	0.0009 (6)
C4	0.0223 (8)	0.0294 (8)	0.0189 (8)	−0.0003 (6)	0.0085 (7)	−0.0058 (6)
C5	0.0218 (8)	0.0218 (8)	0.0272 (8)	−0.0022 (6)	0.0134 (7)	−0.0072 (6)
O1	0.0285 (6)	0.0209 (6)	0.0233 (6)	−0.0069 (5)	0.0091 (5)	0.0011 (4)
O2	0.0226 (6)	0.0232 (6)	0.0196 (6)	−0.0046 (4)	0.0073 (5)	0.0025 (4)
O3	0.0194 (6)	0.0228 (6)	0.0316 (7)	0.0047 (4)	0.0117 (5)	0.0022 (5)
O4	0.0326 (7)	0.0179 (6)	0.0386 (7)	0.0018 (5)	0.0200 (6)	−0.0013 (5)
C6	0.0172 (7)	0.0160 (7)	0.0214 (8)	0.0018 (5)	0.0090 (6)	0.0020 (5)
C7	0.0191 (7)	0.0174 (7)	0.0190 (7)	−0.0002 (6)	0.0105 (6)	0.0006 (5)
C8	0.0208 (7)	0.0154 (6)	0.0180 (7)	0.0004 (5)	0.0104 (6)	0.0016 (5)
C9	0.0192 (7)	0.0155 (7)	0.0189 (7)	0.0015 (5)	0.0088 (6)	0.0005 (5)
C10	0.0219 (7)	0.0187 (7)	0.0147 (7)	0.0020 (6)	0.0076 (6)	−0.0013 (5)

### Geometric parameters (Å, °)

**Table:**

N1—C1	1.3435 (19)	O1—C6	1.2491 (18)
N1—C5	1.364 (2)	O2—C6	1.2774 (19)
N1—H1N1	0.8600	O3—C10	1.3235 (19)
N2—C1	1.338 (2)	O3—H1O1	0.8200
N2—H2N1	0.8600	O4—C10	1.2125 (19)
N2—H2N2	0.8600	C6—C7	1.524 (2)
N3—C2	1.3698 (19)	C7—C8	1.535 (2)
N3—H3N1	0.8600	C7—H7A	0.981 (18)
N3—H3N2	0.8600	C7—H7B	0.98 (2)
C1—C2	1.430 (2)	C8—C9	1.523 (2)
C2—C3	1.378 (2)	C8—H8A	0.999 (18)
C3—C4	1.408 (2)	C8—H8B	0.987 (18)
C3—H3A	0.96 (2)	C9—C10	1.510 (2)
C4—C5	1.353 (2)	C9—H9A	0.98 (2)
C4—H4A	0.96 (2)	C9—H9B	1.00 (2)
C5—H5A	0.97 (2)		
			
C1—N1—C5	123.94 (14)	O1—C6—O2	122.93 (14)
C1—N1—H1N1	118.0	O1—C6—C7	119.46 (14)
C5—N1—H1N1	118.0	O2—C6—C7	117.48 (13)
C1—N2—H2N1	120.0	C6—C7—C8	109.78 (12)
C1—N2—H2N2	120.0	C6—C7—H7A	108.9 (11)
H2N1—N2—H2N2	120.0	C8—C7—H7A	110.1 (11)
C2—N3—H3N1	120.0	C6—C7—H7B	109.2 (11)
C2—N3—H3N2	120.0	C8—C7—H7B	110.4 (11)
H3N1—N3—H3N2	120.0	H7A—C7—H7B	108.4 (16)
N2—C1—N1	118.36 (13)	C9—C8—C7	111.52 (12)
N2—C1—C2	123.19 (14)	C9—C8—H8A	109.1 (10)
N1—C1—C2	118.44 (14)	C7—C8—H8A	109.5 (10)
N3—C2—C3	123.32 (14)	C9—C8—H8B	109.2 (10)
N3—C2—C1	119.05 (14)	C7—C8—H8B	109.0 (10)
C3—C2—C1	117.63 (14)	H8A—C8—H8B	108.5 (14)
C2—C3—C4	121.33 (15)	C10—C9—C8	114.57 (13)
C2—C3—H3A	118.8 (13)	C10—C9—H9A	107.5 (12)
C4—C3—H3A	119.9 (12)	C8—C9—H9A	111.5 (12)
C5—C4—C3	119.41 (15)	C10—C9—H9B	107.4 (11)
C5—C4—H4A	119.1 (12)	C8—C9—H9B	109.1 (12)
C3—C4—H4A	121.5 (12)	H9A—C9—H9B	106.4 (16)
C4—C5—N1	119.17 (15)	O4—C10—O3	124.24 (14)
C4—C5—H5A	125.4 (12)	O4—C10—C9	124.27 (15)
N1—C5—H5A	115.4 (11)	O3—C10—C9	111.48 (13)
C10—O3—H1O1	109.5		
			
C5—N1—C1—N2	−177.92 (14)	C3—C4—C5—N1	−0.9 (2)
C5—N1—C1—C2	3.0 (2)	C1—N1—C5—C4	−1.6 (2)
N2—C1—C2—N3	−1.0 (2)	O1—C6—C7—C8	−101.51 (16)
N1—C1—C2—N3	178.07 (14)	O2—C6—C7—C8	74.36 (17)
N2—C1—C2—C3	179.09 (15)	C6—C7—C8—C9	58.61 (16)
N1—C1—C2—C3	−1.8 (2)	C7—C8—C9—C10	175.91 (13)
N3—C2—C3—C4	179.59 (15)	C8—C9—C10—O4	−12.6 (2)
C1—C2—C3—C4	−0.5 (2)	C8—C9—C10—O3	167.48 (13)
C2—C3—C4—C5	1.9 (3)		

### Hydrogen-bond geometry (Å, °)

**Table:**

*D*—H···*A*	*D*—H	H···*A*	*D*···*A*	*D*—H···*A*
N1—H1N1···O2^i^	0.86	1.94	2.7571 (18)	159
N2—H2N1···O1^i^	0.86	2.13	2.9077 (19)	151
N2—H2N2···O1^ii^	0.86	2.04	2.8766 (18)	164
N3—H3N1···O4^iii^	0.86	2.16	3.0054 (18)	168
N3—H3N2···O1^ii^	0.86	2.17	3.0194 (18)	167
O3—H1O1···O2^iv^	0.82	1.74	2.5546 (18)	171

Symmetry codes: (i) *x*, *y*, *z*+1; (ii) −*x*+1, −*y*+2, −*z*+2; (iii) −*x*+1, *y*+1/2, −*z*+3/2; (iv) *x*+1, −*y*+3/2, *z*+1/2.
